# Cognitive Remediation and Emotion Skills Training (CREST) for anorexia nervosa in individual format: self-reported outcomes

**DOI:** 10.1186/s12888-015-0434-9

**Published:** 2015-03-20

**Authors:** Kate Tchanturia, Eli Doris, Vicki Mountford, Caroline Fleming

**Affiliations:** King’s College London, Division of Psychological Medicine, Institute of Psychiatry, SE5 8AF London, UK; Eating Disorders Unit, South London and Maudsley NHS Foundation Trust, London, UK; Illia State University, Tbilisi, Georgia

**Keywords:** Anorexia, Flexibility, Social interaction, Social cognition, Emotion skills, Anhedonia, Alexithymia

## Abstract

**Background:**

To evaluate self-reported outcomes after a brief course of skills-based individual therapy for inpatients with anorexia nervosa (AN).

**Methods:**

In this case series study 37 adults with AN participated in cognitive remediation and emotion skills training (CREST) sessions, and completed social anhedonia, alexithymia and motivational measures before and after the intervention.

**Results:**

The CREST primary outcome measures were total scores on the Revised Social Anhedonia Scale (RSAS), which decreased significantly (p = 0.03) with an effect size of 0.31, and the Toronto Alexithymia Scale (TAS), which also decreased significantly (p = 0.05) with an effect size of 0.35. The secondary outcome measures focused on motivation: perceived ‘importance to change’ and ‘ability to change’; the second of which increased significantly (p < 0.001) with a medium effect size (d = 0.71).

**Conclusions:**

The individual format of CREST led to a decrease in patients’ self-reported social anhedonia, an improvement in the ability to label their emotions, and increased confidence in their ability to change. Considering the limited number of individual sessions, this is a promising preliminary finding which warrants further research.

## Background

Social and emotional difficulties in the anorexia nervosa (AN) adult population are well recognised [1,2]. Additionally, the poor work and social adjustment which accompany chronic AN make recovery difficult [3]. An increasing amount of research is being carried out in order to explore the ways in which people with AN can be helped to return to a normal level of functioning and adjustment. A new wave of psychological interventions in eating disorders which target improving patients’ social life should therefore benefit their overall quality of life, and might also facilitate the process of recovery [4].

Cognitive Remediation and Emotion Skills Training (CREST) was developed with the purpose of addressing emotion processing, to be delivered over 10 sessions in an individual format [4-6]. In a study by Davies and colleagues [5], patients from different inpatient programmes receiving either treatment as usual or CREST were compared in terms of their performance on emotion-processing behavioural (experimental) tasks. It was found that the patients who had received CREST demonstrated a larger magnitude of change in neuropsychological task performance, although the results were not statistically significant. In previous research [6], qualitative analysis showed that CREST was perceived positively by patients, who reported that education regarding the function, management and expression of emotions was useful [6]. A small study on the group format of the CREST intervention [4] found positive small changes in social anhedonia and motivation; furthermore, when patients were asked what they found helpful about the group, they commented on learning new strategies to deal with emotions and being with other people. Preliminary research therefore suggests that CREST might be a valuable initial intervention enabling patients with AN to process their emotions, which may in turn help them to engage in other therapies [6]. Further investigation is required to explore the benefits of CREST in this patient group and to identify the number of sessions sufficient to yield improvements in emotion processing.

Our aim throughout the development of CREST has been to translate updated experimental findings into a manualised 10 session individual treatment package. The aforementioned studies helped us to evaluate the first version of the manual; and feedback from clinicians and patients as well as more recent research findings (e.g. in positive psychology [7], the expression of emotions [8-10], social communication difficulties [11], social anhedonia [12,3], and emotional intelligence [13]) enabled us to revise the manual. We then offered the revised version to inpatients within the adult service and asked them to evaluate its benefits using self-report questionnaires.

The aim of this study was to explore the self-reported outcomes from inpatients with AN who received an intervention which targets emotion skills. This was not done in the previous quantitative and qualitative studies evaluating the CREST manual. Specifically, we were interested in exploring whether social anhedonia and alexithymia (the ability to recognise, describe and express emotions), both of which are targeted by CREST, showed improvements after 10 individual sessions of therapy. Social anhedonia and alexithymia were chosen as targets due to the difficulties in these areas experienced by patients with AN, which has been demonstrated extensively by research [3,12]. CREST provides psycho-education on the research findings from the studies mentioned above, as well as a variety of skills based, concrete exercises which are tailored to suit the specific needs of the patients. Some of these exercises involve the following: helping patients to name their emotions and feelings (e.g. by giving them a list of emotion words and encouraging them to think about how they feel at that particular moment), encouraging patients to share and express their emotions in a safe way, supporting them to find good strategies around being with other people and communicating their emotional states (e.g. by stating what they think or how they feel), increasing their awareness of pleasurable hedonic experiences (e.g. by generating lists and sharing readily available hand-outs of lists of pleasurable things which do not involve social eating), and helping them to develop positive biases (e.g. by looking at things from different angles, training the brain to notice 3 positive things every day, and trying to identify what is good about the treatment programme or the people with whom we communicate on the ward).

Overall, the aim of CREST is to normalise emotions via the provision of research evidence and psycho-education as to the nature and function of emotion processing. Through the experiential exercises in sessions and homework tasks, it is hoped that patients will develop a vocabulary for emotional experience which can enable them to become more assertive in meeting their needs.

The format of the intervention is simple, brief, and designed for nutritionally compromised patients in an individual format. The intervention used in the current study overlaps with cognitive remediation therapy (CRT) as it includes specific and very concrete examples and activities to facilitate discussion, with materials based on art, metaphors, and scenarios (for details on CRT exercises and the clinical manual please see the following website: http://www.national.slam.nhs.uk/about-us/our-experts/dr-tchanturia/).

The aims of this study were to investigate whether the individual format of CREST can produce changes on self-reported social anhedonia, alexithymia, and motivation to change measures.

## Methods

### Participants

**Figure 1:**
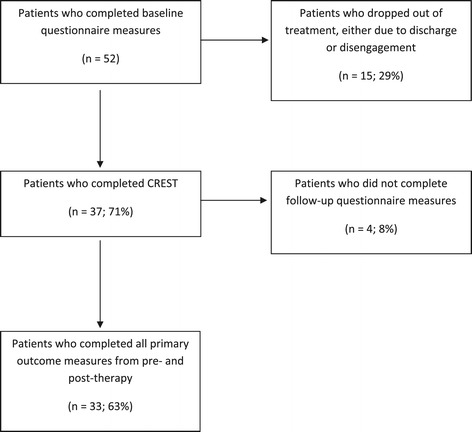
**Consort diagram detailing the collection of self-reported outcome measures for CREST.**

The participants receiving CREST were inpatients from the South London and Maudsley National Adult Eating Disorders service. All patients who had been referred consecutively to the inpatient ward were offered individual CREST. Ethical approval was gained from the National Health Service (NHS) ethics committee (ref: 08/H0606/58). All participants gave written consent, prior to participation in the study. 37 patients were assessed before the CREST intervention started. Of this number, 33 patients (89%) attended all 10 sessions and provided both pre- and post-intervention primary outcome measures. There were some patients in the inpatient programme who received CREST, but did not complete the questionnaires (N = 15) (see Figure 1).

### CREST

Cognitive Remediation and Emotion Skills Training (CREST) is a low-intensity individual manualised treatment for inpatients with severe AN. It aims to target rigid and detail-focused thinking styles (in 1 session), but places greater emphasis on the development of emotion recognition skills (in ourselves and others), and the management and expression of emotion in AN. In CREST, therapists provide updated psycho-educative material (from KT who supervises the therapy and updates the materials with new experimental findings) and facilitate simple, collaborative cognitive tasks and game-like activities that encourage reflection on emotion processing skills (for example, by presenting balls with emotion expressions and discussing what it is like to be sad, happy, or curious) as we have found this is more conducive to discussion than is the approach of asking open ended questions. Therapists also encourage patients to practice implementing small behavioural changes (e.g. noticing and saying what went well and letting their key nurses know that “I felt supported today” or “I enjoyed group walk”). Different modules within CREST are designed to help individuals learn about a) the function of emotions, b) how to label and identify emotions in oneself and others, c) the positive intentions of emotions and the needs emotions communicate to the self and others, and d) tolerating and expressing emotions. The intervention is based on the cognitive interpersonal model, and has been adapted to address additional emotional processes following empirical reviews of the existing literature which highlighted what the main areas of emotional difficulty appear to be for people with AN. It was also informed by service user, carer, and clinician input regarding which aspects of emotion processing were perceived to be the most difficult and necessary areas for intervention [14].

### Assessments

The first primary outcome measure of this study, the *Revised Social Anhedonia Scale* (RSAS), assesses the reduced ability to experience social pleasure [15]. Higher scores on the RSAS indicate greater discomfort in being with other people. The scale is comprised of 40 items measured by ‘true’ or ‘false’ statements. Examples of items from the RSAS include: ‘I prefer watching television to going out with other people’ and ‘I attach very little importance to having close friends’. This measure has been used in the eating disorder patient population several times [3,12]. In one study [12], researchers found a mean score of 16.2 among their group of 105 AN patients, while the corresponding value among 136 non-eating disordered controls was 6.1. Similarly, another study [3] reported a mean score of 16.4 among 72 AN patients, whereas their non-eating disordered group had a mean score of 5.5.

The *Toronto Alexithymia Scale* (TAS) [16] is the second primary outcome measure of this study. It consists of 20 items and three subscales assessing difficulties in identifying feelings (e.g. ‘I am often confused about what emotion I am feeling’), difficulties in describing feelings (e.g. ‘It is difficult for me to find the right words for my feelings’), and degree of externally-oriented thinking resulting in a preoccupation with the details of external events (e.g. ‘I prefer talking to people about their daily activities rather than their feelings’). This is the most widely used measure of alexithymia. A score of less than 51 indicates non-alexithymia, equal to or greater than 61 indicates alexithymia, and 52–60 indicates possible alexithymia. The TAS-20 has been found to have good internal consistency for the total score (a = .81), acceptable internal consistency for the subscale scores, and good test-retest reliability (p < 0.01) [16].

A motivational ruler was also administered and includes two questions which explore beliefs about the *importance* to change and perceived *ability* to change. The questions are answered on a Likert 0–10 scale. Higher scores indicate more positive beliefs about one’s importance/ability to change.

### Statistical analysis

SPSS version 21 was used to analyse the data.

**Figure 2:**
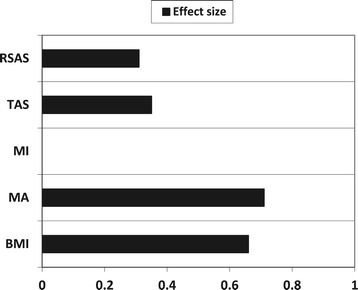
**Effect sizes of the primary self-report outcome measures (RSAS and TAS total score) and the secondary outcome measures (Importance to change, Ability to change, and BMI), post-therapy.**

Pairwise t-tests were conducted to explore differences in pre- and post-intervention levels of social anhedonia, alexithymia, motivation and BMI. Cohen’s *d* (mean_1_-mean_2_/pooled standard deviation) was calculated to provide effect sizes for normally distributed data, with an effect size of <0.2 defined as small, ≥0.5 defined as medium and ≥ 0.8 defined as large. The results are presented in Figure 2.

## Results

### Group characteristics

The mean age of the participants was 24.5 years (SD = 8.2), and the range was 18–54 years. The average duration of illness (measured at the start of CREST) was 8 years (SD = 7.2). The mean BMI at the start of therapy was 15.1 (SD = 1.95).

### Outcome measures

RSAS and TAS total score, motivation (both perceived ‘importance to change’ and ‘ability to change’), and BMI were measured at pre- and post-intervention (the time between assessment before and after CREST was 10 weeks on average). These values along with those from the t-tests conducted are displayed in Table 1.

**Table 1:** **Primary and secondary outcome measures at the beginning and end of therapy**

**Outcome measures**	**Pre-intervention**			**Post-intervention**			**p**	**d**
	**N**	**Mean**	**SD**	**N**	**Mean**	**SD**		
**RSAS**	*34*	*15.3*	*7.49*	*34*	*12.9*	*8.13*	*0.03*	*0.31*
**TAS**	*33*	*63.1*	*9.18*	*33*	*59.8*	*9.79*	*0.05*	*0.35*
**Importance to change**	*30*	*7.87*	*2.71*	*30*	*7.87*	*2.69*	*1.00*	*0.00*
**Ability to change**	*30*	*4.66*	*2.47*	*30*	*6.29*	*2.16*	*0.00*	*0.71*
**BMI**	*37*	*15.1*	*1.95*	*37*	*16.4*	*2.06*	*0.00*	*0.66*

RSAS and TAS total scores were found to have decreased significantly following the intervention, with small effect sizes. With regard to motivation, perceptions of ‘importance to change’ did not differ significantly between pre- and post-intervention, but ‘ability to change’ increased significantly after CREST, with a medium effect size. BMI also increased significantly between the beginning and the end of therapy, with a medium effect size (see Figure 1).

## Discussion

The aim of this exploratory study was to evaluate the efficacy and impact of a novel and brief skills-based therapy for inpatients with anorexia. Recent research findings have highlighted that abstract and flexible thinking styles seem to be less present in individuals with AN [17,3], despite other cognitive strengths such as high IQ [18] and good working memory [19] remaining intact. Eating disorders research has also made progress in characterising low emotional intelligence [13], poor positive emotional expression [8-10], difficulties with social communication, difficulties in friendships [11], emotionally-driven thinking as ‘hot’ cognition [20,21], high social anhedonia [3,12], difficulties with social interactions [22,11], and the patterns of both positive [7] and negative [23] emotion processing. Qualitative assessments of patients’ emotional needs [14], along with these recent research developments, have been translated to inform treatment interventions such as cognitive and emotional remedial therapies; for example, CRT [24,25] and CREST [5].

This study is the first of its kind in that we have explored the feasibility of delivering CREST in a short (10 sessions), individual format using self-report measures of social anhedonia and alexithymia. We found a clinically significant decrease in social anhedonia and alexithymia among the participants, and these aspects were directly targeted by CREST. BMI and motivation were not directly addressed in CREST, but were used here as secondary measures to assess improvements in other important domains. The results show that the main outcomes, such as improved use of emotional vocabulary and development of the ability to be with other people, changed in the positive direction. Small effect sizes (0.31-0.35) and statistical significance together with patients’ comments on how they benefited from CREST suggest that CREST is a useful tool for this severe group of patients receiving inpatient treatment.

We are aware of several limitations within the current study and will try to improve upon these shortcomings in future research and evaluation. For example, we would like to explore in more detail exactly who benefits the most from this intervention and audit more precisely the clinical characteristics of patients who either do not respond to or do not choose to take part in this therapy. It would be desirable to explore the presence of autistic characteristics within this group of patients, since the current research findings suggest that many inpatients with AN have elevated levels of autistic traits [26]. In summary, this study provides some evidence that our brief CREST intervention can influence social anhedonia, the ability to recognise one’s own emotions, and confidence in one’s ability to change. These findings are promising because from previous research we know that social anhedonia is highly correlated with chronicity of illness and the current data has been collected from adult patients with a long duration of illness (the mean duration of illness in this study is 8 years). However, further research is needed in order to corroborate these findings; to be precise, a randomised controlled design study with a larger sample size.

### Strengths

This is our attempt to contribute towards intervention developments targeting emotion skills in severely nutritionally compromised patients with AN. The intervention has been positively received by patients and therapists, and the manual has been informed by the latest research updates. Well-tested outcome measures which seem to tap into problematic areas for eating disorder patient groups have been selected, which also capture changes after CREST.

### Limitations

The absence of accurate information explaining the reasons for ‘drop out’ cases (e.g. early discharge, not wanting to complete questionnaires) is a clear limitation of this study.

Further improvements of this study would include addressing the limitation mentioned above and refining our assessment battery to include tests measuring emotion expression, an area into which CREST taps and which has been shown to be sub-optimal in people with AN [8,9].

## Conclusion

Promising results from the self-report measures combined with meaningful feedback from the patients participating, leads us to the conclusion that it would be worthwhile to further develop this line of clinical research.
